# Action research and health system strengthening: the case of the health sector support programme in Mauritania, West Africa

**DOI:** 10.1186/s12961-020-0531-1

**Published:** 2020-02-19

**Authors:** Kirsten Accoe, Bruno Marchal, Yahya Gnokane, Dieng Abdellahi, Paul Bossyns, Bart Criel

**Affiliations:** 1 Department of Public Health, Institute of Tropical Medicine, Nationalestraat 155, 2000 Antwerp, Belgium; 2 AI-PASS Programme (Institutional Support for Health Sector Strengthening), Enabel – Belgian Development Agency, Nouakchott, Mauritania; 3 Department of Health, Enabel – Belgian Development Agency, Rue Haute 147, 1000 Brussels, Belgium

**Keywords:** Health system strengthening, Action research, Learning organisation, Participatory approach

## Abstract

**Background:**

Access to qualitative and equitable healthcare is a major challenge in Mauritania. In order to support the country’s efforts, a health sector strengthening programme was set up with participatory action research at its core. Reinforcing a health system requires a customised and comprehensive approach to face the complexity inherent to health systems. Yet, limited knowledge is available on how policies could enhance the performance of the system and how multi-stakeholder efforts could give rise to changes in health policy. We aimed to analyse the ongoing participatory action research and, more specifically, see in how far action research as an embedded research approach could contribute to strengthening health systems.

**Methods:**

We adopted a single-case study design, based on two subunits of analysis, i.e., two selected districts. Qualitative data were collected by analysing country and programme documents, conducting 12 semi-structured interviews and performing participatory observations. Interviewees were selected based on their current position and participation in the programme. The data analysis was designed to address the objectives of the study, but evolved according to emerging insights and through triangulation and identification of emergent and/or recurrent themes along the process.

**Results:**

An evaluation of the progress made in the two districts indicates that continuous capacity-building and empowerment efforts through a participative approach have been key elements to enhance dialogue between, and ownership of, the actors at the local health system level. However, the strong hierarchical structure of the Mauritanian health system and its low level of decentralisation constituted substantial barriers to innovation. Other constraints were sociocultural and organisational in nature. Poor work ethics due to a weak environmental support system played an important role. While aiming for an alignment between the flexible iterative approach of action research and the prevailing national linear planning process is quite challenging, effects on policy formulation and implementation were not observed. An adequate time frame, the engagement of proactive leaders, maintenance of a sustained dialogue and a pragmatic, flexible approach could further facilitate the process of change.

**Conclusion:**

Our study showcases that the action research approach used in Mauritania can usher local and national actors towards change within the health system strengthening programme when certain conditions are met. An inclusive, participatory approach generates dynamics of engagement that can facilitate ownership and strengthen capacity. Continuous evaluation is needed to measure how these processes can further develop and presume a possible effect at policy level.

## Background

Mauritania is a country in the Maghreb region of North-western Africa. A substandard healthcare system is one of its biggest challenges, leading to inadequate accessibility to equitable healthcare of good quality. An audit within the Ministry of Health in 2014 attributed the weak governance and performance of the health system to excessive centralisation, system fragmentation, lack of programme coordination, poor efficiency in terms of resource allocation and lack of technical competencies [1]. In order to address these weaknesses, a 4-year programme, known as “*Appui Institutionnel au Programme d’Appui au Secteur de la Santé*” (AI-PASS; i.e., Institutional Support for Health Sector Strengthening), funded by the European Commission, was launched in July 2017 [2]. The programme is implemented by Enabel (the Belgian Development Agency) and supported by the Public Health Department of the Institute of Tropical Medicine (ITM). It aims at assisting the Ministry of Health in the implementation of its national development plan and encompasses five domains, namely governance, equitable access to quality healthcare, essential drugs and consumables, human resources management, and setting up a social health insurance scheme. To improve exchange and dialogue within the national health system and enhance its responsiveness to people’s needs, the programme has a two-fold approach – to provide institutional support at the central level and guidance to two health districts at operational level.

In essence, the AI-PASS programme is about health system strengthening. The rise of global health initiatives has increased the need for a comprehensive systems perspective [3, 4]. Yet, in many countries, health system strengthening approaches still focus on narrow aspects of the health system. The literature shows that there is a knowledge gap regarding enhancing health system performance in low- and middle-income countries. Not only is there little documented and comprehensive information on the performance of health systems and on how policies affect performance, but policy-makers are often not aware this information exists or are not using it in an adequate manner [5, 6]. There has been, for example, little systematic consideration in the literature of the extent to which multi-stakeholder efforts in processes actually give rise to changes in health policy [7]. Health systems are best considered as complex, adaptive systems, characterised by non-linearity, feedback loops and path dependency [4, 8, 9]. Managing and changing complex systems requires participatory initiatives, piloting of innovations and continuous evaluations to better deal with their inherent uncertainty. Developing such learning systems demands research embedded in local realities [10] and the wide use of systems tools [3]. Embedded health policy and systems research (e-HPSR) fits this bill. One of the core characteristics of e-HPSR is the systematic use of research methods to improve policies, programmes, local healthcare systems and knowledge translation by applying the gained knowledge in real time to initiate change [11]. e-HPSR provides multiple benefits. It addresses relevant real-world questions as defined and stated by local (health) actors, it increases the likelihood of ownership and accountability because it closes the gap between research and policy, it is responsive to community needs, and it can improve the sustainability of change within the system by developing a learning system [10, 12].

Within the AI-PASS programme, action research plays a central role. Koshy [13] defined action research as a method for improving practice; it involves action, evaluation and critical reflection. Based on cumulative evidence, changes in practice will be implemented and evaluated. Action research is built on four fundamental pillars – (1) empowerment of local actors, (2) participative collaboration, (3) acquisition of knowledge, and (4) social change through action. Action research thus serves a double purpose – it induces change within the healthcare system (‘action’) and it generates knowledge at local, national and international levels (‘research’) [14]. Action research can thus be considered as an e-HPSR approach.

**Fig. 1:**
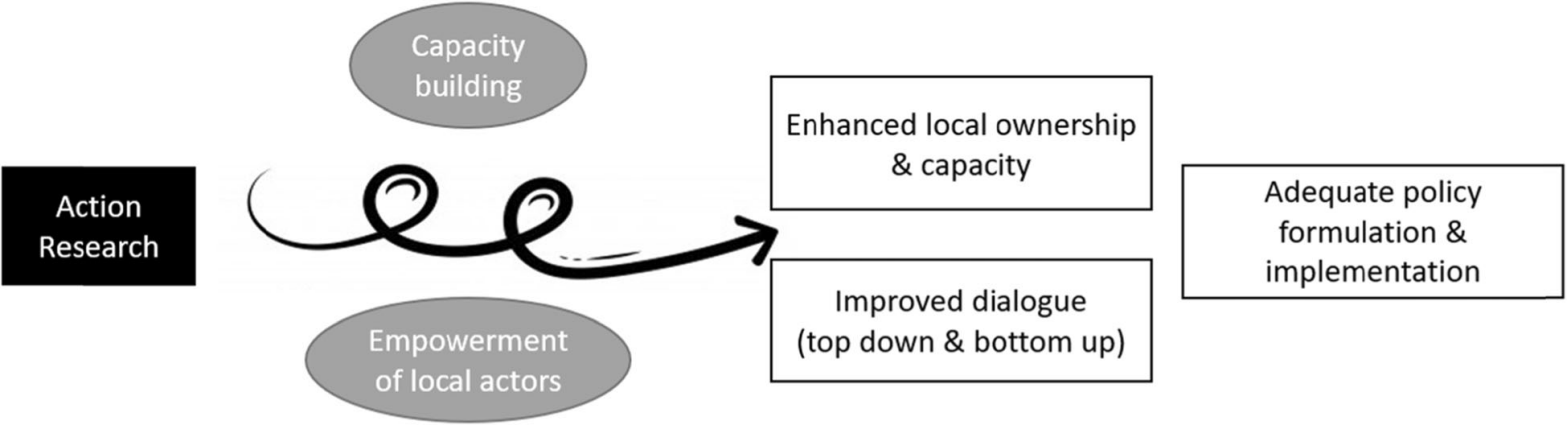
Defined hypothesis of the possible impact of the action research component of the AI-PASS programme

The choice for action research as a central element of the AI-PASS programme was based on the hypothesis that an inclusive, participatory and cyclical approach to problem identification and solution analysis might contribute to sustained change at the local level, and that the lessons learned might inform regional and national policies. The expected changes include enhanced local ownership and capacity and an improved dialogue between the different levels of the health system, which would contribute to an adequate process of policy formulation and implementation within the health system. In practice, the action research process had two main elements, namely (1) an in-depth local health system (LHS) analysis and (2) the identification, development and implementation of capacity-building and empowerment activities. The action research component would act through the latter two mechanisms, that is capacity-building and empowerment of local actors (Fig. 1).

In this paper, we describe the results of an evaluation of the action research approach in the AI-PASS programme. The objective of this study was to describe and analyse the ongoing participatory action research programme in Mauritania and, more specifically, to see in how far action research, as an embedded research approach, could contribute to strengthening health systems.

## Methods

### Study design

We adopted a single-case study design and defined the ‘case’ as the health system strengthening approach of the AI-PASS programme in Mauritania. We chose two sites, namely the learning districts (“*Moughataas d’Apprentissage*”) of Dar Naim and Bababé. These districts were selected as AI-PASS sites on the basis of ITM’s experience and presence in Mauritania. There was a deliberate choice for one rural and one urban area in order to support the Ministry of Health in the implementation and adaptation of its national strategies in rural and urban zones. Both districts indeed present the deficits of the weak governance and low performance of the health system. Further details are described in Additional file 2.

The study population of our case study included representatives of the communities and civil society of the two districts and the local, regional and central actors in the Ministry of Health of Mauritania.

The case study had the following components: (1) a description of the development of the AI-PASS programme; (2) a description and assessment of the LHS analysis that was carried out in each district; (3) a description and assessment of the capacity-building and empowerment activities conducted in these two districts as a result of the situation analysis; and (4) identification and assessment of the effects of the action research approach.

The study period was from mid-2016 to March 2019.

### Study setting

Mauritania comprises a territory of more than 1 million km^2^, with about 4.3 million inhabitants and a low population density. For a decade now, internal migration has increased towards the capital, Nouakchott, where one-third of the population lives. Mauritania has substandard healthcare and problems of geographic inaccessibility. The maternal mortality ratio was estimated at 582/100,000 live births and infant mortality was estimated at 72/1000 live births in 2013 [15]. Healthcare is costly; the out-of-pocket expenditure (out of total health expenditure) is relatively high, at 43% [16]. This considerable cost of healthcare affects the vulnerable populations most, leading to catastrophic health expenditure and increased impoverishment. Currently, 42% of the population (59.4% rural, 20.8% urban) lives below the poverty level and 25.9% (40.8% rural, 7.7% urban) lives below the extreme poverty level [17].

### Data collection

Three methods were used to collect qualitative data.

#### Document review

We carried out a document review to collect relevant national policy and programme documents, field visit reports, meeting summaries and workshop results.

#### Interviews

We conducted semi-structured interviews with key informants. To identify key informants, we used purposive and opportunistic sampling techniques. The selection was based on each informant’s current position and participation in programme activities (Table 1). After 12 interviews, data saturation was achieved. Written informed consent was obtained from all participants during the semi-structured interviews. Interviews were recorded when participants consented. Interviews lasted between 30 and 55 min, and they were conducted in French. An interview guide was elaborated (Additional file 1) and adapted iteratively during the process.

**Table 1:** Characteristics of interviewees

Characteristic	Number of interviewees
Central level MoH	2
Regional level MoH	1
Local level MoH	1
Local level – civil society representative	3
Local level – community representative	1
Members of the AI-PASS programme team	4
Total	12

*MoH* Ministry of Health, *AI-PASS* Institutional support for health sector strengthening

#### Participatory observation

We engaged in participatory observations of meetings, workshops and training sessions, in which local actors and representative authorities (at the local, regional and central levels of the Ministry of Health) participated. All participants were informed orally about the study.

We collected data for each step of the case study (Table 2).

**Table 2:** Sources of data for analysing the health system strengthening approach of the AI-PASS programme in Mauritania (mid 2016 – March 2019)

Objectives	Data-collection methods	Sources
Description of the development of the AI-PASS programme	Document review	Programme and policy documents
		Minutes of meetings with Mauritanian and Belgian partner institutions (2017–2018)
		Initial field visit reports (2017)
Assessment of the local health system analysis	Document review	Reports on district analyses (2018)
		Research notes from meetings and discussions (2018)
		Field visit reports by the first author (2018–2019)
Capacity-building and empowerment	Document review	Minutes of meetings with national experts (2018–2019)
		Presentations and reference documents on various concepts of a local health system and action research (2018–2019)
		Presentations on the progress of the district analysis, prepared by the national experts and district health officers (2018)
	Participatory observation	Observation notes (2018–2019)
	Semi-structured interviews	Transcripts and research notes from 12 interviews (2019)
Effects: enhanced local ownership and capacity, improved dialogue, and adequate policy formulation and implementation	Document review	Summary reports of panel discussions during the October 2018 workshop
		Minutes of meetings with Mauritanian and Belgian partner institutions (2018–2019)
		Minutes of meetings of AI-PASS programme staff (2018–2019)
		Minutes of meetings with the action research Steering Committee (2018–2019)
	Semi-structured interviews	Transcripts and research notes from 12 interviews (2019)
	Participatory observation	Observation notes (2018–2019)

*AI-PASS* Institutional support for health sector strengthening

### Data analysis

The first recorded interviews were transcribed verbatim by the first author (KA). The remaining interviews were transcribed by an independent translator. All were checked for accuracy by the first author. The interviews were then entered into NVivo 12 software for data management and analysis.

We used a thematic coding approach to analyse the primary data. Data from the document review, capturing the capacity-building activities and identified changes, were entered into a NVivo 12 project for analysis. Coding and thematic analyses were carried out by the first author (KA) and checked for accuracy by the last author (BC). An initial coding tree was elaborated deductively, based on our hypothesis and the objectives of the study. The coding tree evolved during the analysis. When we categorised the common elements in the interview transcripts and documents (meeting minutes and visit reports), diverse topics and patterns emerged. Recurrent themes included pathways of change, challenges, identified barriers and recommendations.

Reflections of workshops and observations of meetings and trainings were systematically collected in a separate Excel file. By analysing the notes taken by the first author and by discussing experiences with the team members of the programme, these data were used for triangulation.

### Ethical considerations

We applied for and received ethical approval from the Institutional Review Board of ITM (Ref N° 1280/19). We received study approval from the Ministry of Health of Mauritania (Ref N° 003/2019).

## Results

### The development of the AI-PASS programme

A Mauritanian non-governmental organisation, known as the *Association pour la Promotion de la Santé de Dar Naim* (APSDN), has been involved in the districts of Dar Naim (since 1998) and Bababé (since 2008) with support from the Belgian non-governmental organisation Memisa [18–21].

Building on this long-term involvement in the field, the *Programme d’Appui au Secteur de la Santé* (a Support Programme for the Health Sector) was developed in collaboration with Enabel and with funding from the European Union.

To improve exchange and dialogue within the national health system and to enhance its responsiveness to people’s needs, the project adopted a two-fold approach, providing institutional support at central level, combined with follow-up of the management of two health districts, Bababé and Dar Naim. This integrated top-down and bottom-up methodology, known as “*double anchoring*” [2], is a central approach used within the development programmes of Enabel. This strategy aims to stimulate regulation and encourage setting standards, on the one hand, and to systematically identify the lessons learned to enrich policy-making, on the other. The objective was to enhance the autonomy of the two district health teams by guiding them towards an improved performance of their LHS through action research cycles. Concomitantly, accountability to the population was emphasised. An adequate dialogue between the health staff and the community was imperative to ensure strategies were tailored to local needs.

**Fig. 2:**
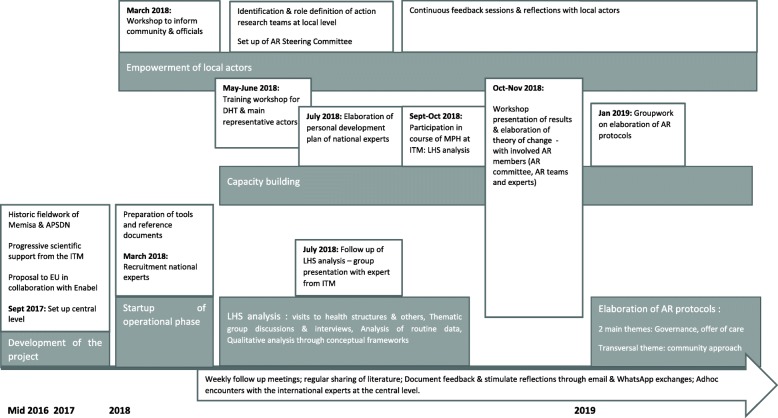
Timeline of the AI-PASS programme, from its inception in mid-2016 to March 2019, with the main events and activities. *APSDN* Association pour la Promotion de la Santé de Dar Naim, *AR* action research, *DHT* district health team, *EU* European Union, *ITM* Institute of Tropical Medicine, *LHS* local health system

Figure 2 provides an overview of the process of the AI-PASS programme from its initial conception in 2016–2017 to March 2019. Key events and main inputs in terms of capacity-building and empowerment of local actors are presented.

The action research team consisted of ‘operational’ and ‘external’ researchers*.* Given the diverse backgrounds of the actors involved, constant discussions were needed to build a common vision. Operational researchers were the district health officer, the district health team, health professionals and the beneficiaries, who served as community representatives and members of civil society. They contributed to the research through their extensive knowledge of the local context and were considered of key importance in the implementation of the identified priorities. External researchers contributed to the research through their experience and extensive knowledge of public health-related topics. They included the staff members of the AI-PASS programme and experts from the three partner institutions. The regional and central directors were the authorities who had to be informed regularly, could offer support, and would have had to give the ‘green light’ for decisions made at the local level. The degree of their involvement varied – the two national staff members (hereafter called national experts) were recruited by the programme to perform close follow-ups of the processes in the two districts.

The action research Steering Committee, which comprised central and regional health directors, was created to enhance national ownership and to facilitate the inclusion of innovative practices into policy-making. The committee met once per trimester. The operational researchers met initially on an ad hoc basis but, after November 2018, they met on an increasingly regular basis.

### The local health system analysis

A first step in the action research approach consisted of a thorough situation analysis of the two selected districts; this was based on the dynamic health system framework [4, 22]. This framework broadened the six building-block model proposed by WHO [5, 23], specifying a central role for the population to increase responsiveness and public accountability, clearly accounting for context, and making underlying values explicit. Finally, it highlights the importance of social determinants of health.

The framework was applied in eight steps (Table 3). In the two Mauritanian learning districts, data were retrieved from documentary reviews (national policy documents, survey results, legal texts, etc.), routine data from the Health Information System, reports from field visits to various health and supporting structures, and discussions with local actors.

**Table 3:** Steps and components of the local health systems analysis and results (capacity-building needs)

Steps of the analysis	Essential components to be evaluated	Results: Defined capacity-building needs within the two districts
1. Description of the local health system	Context (geographic, sociodemographic, socioeconomic) Coverage of health facilities HRH Stakeholder analysis and mapping	Definition of an adequate coverage plan of health services within a district (difference between urban and rural district) Coordination capacity with a multitude of actors in Dar Naim Capacity to assess HRH needs
2. Analysis of interactions between the demand, need and supply dimension of healthcare services	Evaluation of the offered package of activities, compared to the demand from the population and the needs defined by health actors	Definition of a comprehensive integrated package Capacity in terms of mental health, basic obstetric care, etc.
3. Analysis of accessibility and quality of care	Analysis of accessibility by evaluating the existing data (e.g. data provided by the Health Information System) and framework [24] Analysis of quality of care in four dimensions: cost-efficiency, continuity of care, integrated care and patient-centred care [25]	Capacity in data analysis and monitoring and evaluation Knowledge of and experience in patient-centred care General capacity to enhance quality of care (among all health actors)
4. Pharmacy management	Analysis of the process: from supply to management to prescription	General knowledge on pharmacy management Provision of tools
5. Evaluation of local healthcare financing arrangements		Provision of tools Awareness on transparency Knowledge on health insurance and social protection schemes
6. Analysis of the stewardship function of the local health system	Evaluation of the various functions of a DHT [26]; evaluation of the team’s interactions with local, regional and central actors	Leadership skills General public health knowledge according to defined functions of a DHT [26]: Response to local needs in terms of healthcare, coordination of actors, management of health services, activities and health staff, supervision and training, adaptation of national strategies to local context Capacity to analyse health information data
7. Analysis of community participation	Analysis of formal and informal health committees; evaluation of the level of community participation [27, 28]	Awareness on advantages of a genuine partnership and a non-utilitarian vision of community participation Capacity to define a strategy adapted to an urban context (Dar Naim)
8. Synthesis and lessons learned		Capacity to prioritise A systems vision

*DHT* district health team, *HRH* human resources for health

The analysis was performed in each district by the district health officer and the national expert. Additionally, community members (in Bababé) and members of the civil society (in Dar Naim) were involved; the analysis took about 6 months (May to October 2018). Several workshops and an intensive follow-up process were organised in order to become familiarised with the analytical frameworks. Table 3 presents the steps and components of the LHS analysis and its results in terms of identified capacity-building needs. A summary of the results of the analysis is presented in Additional file 2.

### Capacity-building and empowerment

We found that, during the project, capacity-building activities were initially identified during the situation analysis, finetuned throughout, and conducted with the local teams of Dar Naim and Bababé. A detailed timeline is shown in Fig. 2. The activities can be categorised as follows: (1) discussions with local staff, conducted either face-to-face during field visits or through Skype; this was complemented with reading and discussions of documents and scientific papers; (2) development of training sessions based on knowledge gaps identified in the LHS analysis (Table 3); (3) elaboration of personal development plans for the national experts; (4) co-creation and organisation of workshops (LHS, Theory of Change, specific action research protocols); and (5) participation of four Mauritanian health cadres in the 4-week LHS component of the ITM Master’s in Public Health course (academic year 2018–2019).

Our analysis shows that empowerment was encouraged through (1) the promotion of teamwork and dialogue by group exercises and joint reflection sessions with the district health team and local actors:

“*It is usually the doctor that decides; others often don’t have the opportunity to speak, or they don’t dare to express any criticism. But here* [refers to the workshop]*, I have the impression everyone was on the same level. There was a good exchange.*” (Interview 1)

(2) Enhancing accountability by discussions about the need to strengthen individual responsibility and the agency of health workers and the need to implement structural reforms:

“*The most important thing is that local staff, with the support of the community, now identified and proposed possible solutions at local level*.” (Interview 2)

(3) Creation of a safe space to interact, discuss and learn through inquiry and dialogue, where participants were encouraged to express their opinions and thoughts:

“*The debates and discussions made it possible to reflect on sensitive topics, such as the poor communication between the various stakeholders, the difficulty of auto-criticism and political and socio-cultural influences.*” (Interview 4)

(4) Invitation of central and regional actors to workshops in order to enhance comprehension and dialogue with local actors.

### The effects

#### Enhanced local ownership and capacity

Our observations revealed that various community and local health actors showed interest in contributing to the process. When interviewed, they stated that this was the first time they had participated in an exercise in which their opinion was asked. The exchange of experience and mutual enrichment were mentioned as valuable outcomes.

“*Before, people disappeared directly after a workshop. But this time, because they were so interested, they asked for a follow up meeting …*” (Interview 5)

“*I sense that person x has evolved tremendously … in his way of tackling problems, in his understanding of the system organisation.* […] *In the beginning, he frequently criticised our input: there was too much emphasis on negative elements… But, by the end of the second workshop, he was more objective….*” (Interview 10)

The participatory methodology of the action research approach was highly appreciated and enhanced ownership among the participants.

Despite the diversity in competence and experience among the participants, one respondent stated that there was complementarity and support among them. The workshops allowed participants from the community to gain insights in the organisation of LHS but also gave them confidence.

“*… those who participated during the two-week workshop, I’m sure that if they would become part of a health committee now, they wouldn’t have the same attitude. They would be better prepared, and able to share their opinion…*” (Interview 10)

Many participants from the local level indicated the gained confidence by participating in the situation analysis exercise.

“*In the beginning, I didn’t have the tools; I found the methodology difficult and complex … but little by little, through the workshops, training, and discussions, … things became clearer.*” (Interview 5)

“*I don’t know which ‘marabou’ you used, but people were there; they were passionate in expressing their voices, to show what they could do, to look for solutions…*” (Interview 8)

The analysis of data from the document review and the observation notes revealed that the provision of a voice to the community empowered them to speak out and demonstrate against malpractices in one of the districts. As a consequence, authorities were obliged to undertake remedial action.

Some respondents said that the lack of engagement or involvement of health committee members in health matters was the main reason why the committees failed to function. Therefore, the implication of including the community had a double message. One respondent interpreted this as an act of transparency towards the public, but he found it was also a message to the authorities and health staff that demonstrated the added value of involving the community.

“*Someone who is involved from the start acts differently compared with someone brought in when things are already put into practice.*” (Interview 9)

Nevertheless, despite the participatory approach within the action research team, it remained difficult to enhance participation in the daily management of the district. Observation notes showed that community representatives were implicated in the organisation of campaigns or hygiene activities, but little in strategic or financial decisions.

The participation of the two district health officers and the two national experts in the LHS module of the Master’s in Public Health at ITM was one of the most important triggers in their learning processes (source: observation notes and document review). The interviews showed that, initially, they perceived the analytical approach as complicated; however, in time, they felt more comfortable.

Performing the analysis together with the local actors was seen as a form of capacity-building and a way to enhance ownership. The intensive coaching throughout the process was considered important for creating change (source: observation notes and document review). Nevertheless, further observation revealed that some reluctance and a decrease in engagement emerged among certain actors at later stages of the project. Initially, the district health officer expressed clear intentions to improve the organisation of the health services through the organisation of weekly management meetings or initiating delegation of clinical and managerial tasks to nurse-practitioners and other health cadres. We later observed that these plans would be implemented partially or not at all. While some local health staff were highly motivated in the beginning, some backed out after some time. Reasons given for this during the interviews include a lack of follow-up or support and feasibility problems of the identified activities. Indeed, our document review showed that, while the LHS concepts seemed easy to grasp, the actual identification and implementation of clear and comprehensive strategies, priorities and evaluation methods proved difficult. The nonlinear planning and more cyclical approach was questioned at times. The absence of quick results was, for some respondents, a reason to explain the reduced enthusiasm for continuing to engage in the action research cycle (source: observation notes and document review). Lastly, observation of meetings revealed additionally that, for some, the lack of financial gain was quite an important barrier to enhance or maintain engagement.

#### Improvement in dialogue between actors at different levels of the health system

During the meetings and workshops, we observed that there was effectively a dialogue between actors at local level but far less between staff at local and central level.

In one of the districts, a WhatsApp group was created to allow dialogue between community representatives and the district health team, which facilitated the preparation and follow-up of the activities. In the urban district, an improved dialogue was observed between the public and private sector.

Our interviews showed that respondents considered it useful to create a dialogue between the central and the operational level of the health system and to stimulate participation at all levels. While the informants at local level thought it is important that such dialogue ensures that everyone can provide inputs and that decisions are not imposed, it also became clear that organising round tables involving actors from various levels was not easy. Due to busy schedules at higher level and different priorities, active involvement throughout the workshop was difficult. People tended to walk in and out, challenging the atmosphere during the group discussions.

Other constraints were cultural aspects, poverty and an organisational climate not conducive for open communication between levels of hierarchy (source: observation notes and document review). Respondents identified fear of change and concerns about losing personal gain as some of the main inhibitors. The hierarchical structure and power differences were perceived as additional barriers.

“*The main problem is that people think: if I involve him too much, I’ll lose my power.*” (Interview 10)

“*People are afraid of change … there are always the privileged ones, those given priority and advantages; those people are afraid to lose their privileges.*” (Interview 9)

“*There will be a lot of challenges, because the system has worked like that for years … A lot of managers will not see the point of changing anything.*” (Interview 8)

#### Policy formulation and implementation

As described in the Background section, one of the objectives of the project was to ensure adequate and responsive policy formulation and implementation at the central level of the Ministry of Health towards the operational or district level. One proposed strategy was the occasional involvement of district-level staff in national-level meetings. However, we observed that local actors and the project’s national experts were not involved in technical meetings at central level. During interviews, the strong emphasis on the hierarchical structure was identified as a barrier to such an interaction.

Our observations and interviews found a common-held view that the districts were implementers of existing national strategies, rather than co-creators of new ones. An example was the development of a supervision and planification tool that was to be tested at local level, but which was done without any input from the district health management teams. It was also stated that the composition of the district health team should reflect the regional team, even when the latter reinforced rather a vertical approach to management and healthcare delivery and showed little space for innovation. Some respondents indicated that staff at the central level thought that action research required their strong involvement as “*external*” researchers.

“*The vision of our action research was explained, but despite this, we realised … that the central level still did not perceive that action research is, first and foremost, about local actors.*” (Interview 12)

Despite an intensive period of collaboration during workshops or field visits, our observations showed that the engagement from central level staff remained limited over time. One interviewee remarked that this limited ownership at the central level and the difficulty in achieving (pro)active involvement were due to the fact that those individuals were not sufficiently involved in the conceptualisation of the programme and the action research approach. According to this respondent, they did not fully grasp the scope.

“*We realise that, until today, some still do not have a good understanding of action research and what we’re aiming for… We have to find a way to re-explain … and give examples.*” (Interview 12)

Other respondents noted the lack of involvement of the regional departments of health. This concern was also mentioned during one of the debates in October 2018. Respondents stated that the institutional support of the AI-PASS programme at central and local levels should be explicitly expanded to the regional level. On the other hand, our observations and the document review also revealed the various unsuccessful attempts to involve them. Monthly meetings were proposed but only few were held.

## Discussion

The objectives of this study were to investigate whether and how the project’s approach initialised change in terms of responsiveness of the policy formulation and implementation process at central level through capacity-building and empowerment at district level.

Our study showed that the participatory approach and the continuous capacity-building efforts led to higher levels of empowerment at the district level and a better dialogue between local actors. However, these effects seemed fragile. Strong hierarchical structures, current work ethics and broader sociocultural aspects were found to be constraints. The changes expected to take place at the central level – higher efficiency and responsiveness in health policies – were not observed.

We found that the systematic involvement of a range of health actors had a prominent influence on the process of dialogue and empowerment. From a systems perspective, this approach provided an opportunity to break down barriers between patients and providers, and between local actors and local policy-makers [3, 29]. Nevertheless, attempting a participatory approach within a bureaucratic context is difficult. The role of the already mentioned dominant hierarchical organisational culture was also reported by researchers of the District Innovation and Action Learning for Health Systems Development (DIALHS) project in Cape Town, South Africa. Cleary et al. [30] showed that enhanced relational leadership could be blocked by a hierarchical, bureaucratic structure and by pressure from higher levels. In a study on community health worker approaches, Henriksson [31] identified limited decision spaces and inadequate funding as important barriers to using local evidence in the planning process. In a literature review, Gilson et al. [32] described that reasons for exercising power were linked with three types of contextual factors, namely professional norms and practices (e.g. medical hierarchy), sociocultural values (e.g. traditional gender roles or ethnic suppression), and wider political and economic factors (e.g. support from and trust in a government). Cleaver [33] also recommends consideration of the wider dynamics of economic and social change, rather than a focus on a single participatory intervention. Within the scope of our study, these factors were difficult to unravel, but they appeared to play a prominent role. Further socio-anthropological research seems indicated to better understand potential barriers.

We found that the capacity-building approach increased the knowledge of involved local actors. A positive dynamic was observed in the appreciation voiced by local actors and in the motivation to engage in change. This dynamic was consistent with observations by Bossyns and Verlé [34], who stated that action research could enable and motivate stakeholders and that it sought to make them responsible for their acts. Nevertheless, not all actors maintained their initial engagement. Several hypotheses are plausible. Reduced work ethics and a strong focus on extrinsic motivation, possibly fuelled by bilateral and non-governmental organisations and/or due to low salaries, could be one of the reasons. Another explanation could be the weak support and mentoring system at higher levels. These findings can be related to the self-determination theory of Gagné and Déci [35], which defines that intrinsic motivation is easily disrupted and needs support conditions such as feedback mechanisms from superiors and a feeling of relatedness as well as a certain level of autonomy and trust in one’s competences. An equilibrium with financial compensation needs to be found, as the latter can crowd out intrinsic motivation. A study of a nutrition programme in a rural district in South Africa highlighted the importance of a supporting environment for capacity-building through three key elements: tangible and comprehensible knowledge transformation and use of evidence (“*ways of thinking*”), adequate leadership, empowerment and accountability mechanisms (“*ways of governing*”), and adequate inputs and capacity (“*ways of resourcing*”) [36, 37]. Three axes that should be tackled simultaneously and that are, according to our LHS analysis, the main weak factors within the system.

Uptake at central level was challenging and seems to need more time. This could be partially explained by the fact that the flexible iterative process required to manage complex adaptive systems, such as health systems, is difficult to synchronise with the yearly planning cycle of the Ministry of Health [38]. In Mauritania, as in many other low- and middle-income countries, externally designed programmes are dominant. These are often limited in time, focus on one particular health problem or subpopulation, and are geared towards fixed goals framed within donor investments. A systems approach moves away from this linear chain of command [34, 39]. Learning by doing through action research entails a certain level of reasoning, experimenting, analysing and adapting according to the lessons learned [40, 41]. This concept was new for most actors and a certain reluctance was observed during discussions. During the workshops, it was observed that creating a safe learning space gave the participants the confidence to be open to change and reflection.

### Lessons learned for the project

From the current study, we can start formulating some lessons.

To facilitate change, key actors must be acknowledged and actively involved. These pioneers could function as transformative leaders that create trust, communicate a vision and empower others to pursue opportunities. Lipsky stressed the influence of the front-line staff; they should be supported in “*ongoing processes of supportive criticism and inquiry*” [42]. Lehman and Gilson [43] stated that this type of process takes time; one needs to build a relationship, foster trust and adopt flexibility. The cycle of social change described by Prochaska and Velicer stresses the need for a progressive approach that allows all actors the time they need to understand the process, to reflect on it, and to allow trial and error [44].

Advocacy seems essential to maintain the interest of the actors at the central level and should include demonstrating good practices and quick wins. Being confronted with positive changes might enhance the motivation to collaborate and sustain ownership in the long run [45]. Additionally, maintaining constant exchange between the AI-PASS team members is necessary to improve the dialogue within the national health system. The right conditions should be created to guide the ministry progressively towards change, and sufficient time should be provided for discussions and trials. This was also observed in a study that analysed various embedded implementation research projects: engagement of policy-makers at the onset and throughout the research, and actionability (i.e. the relevance of the research to inform a policy) are imperative. An equilibrium is needed between the time and resources required to explore systemic factors and the need to produce actionable results [12]. Documenting this process at the central level could promote further reflection.

### Lessons learned in terms of action research methodology

We found that local actors were essential in identifying problems and solutions, and in acquiring a better understanding of why past attempts to introduce change were not successful [29]. This observation was consistent with the methods described by Loewenson et al. [46], who found that policy-makers could benefit from participatory action research because it provides a wealth of information from those directly involved at the operational level, including individuals that would not otherwise be heard. However, this process must go in both directions. The involvement and collaboration between operational and external researchers can encourage reflection. This was also observed in various programmes that developed similar learning sites, like those in South Africa (DIAHLS) and Kenya (Kilifi), where a collaboration between researchers and health system actors provided opportunities to support managers in taking action and generating knowledge at the same time [29].

Determining the constitution of the research team is a dynamic process. It is quite challenging to stimulate the participation of community representatives and health staff. Linguistic and capacity factors and barriers raised by hidden tribal and cultural customs may hamper or slow down the learning process. The level of participation needs to be seen within a continuum – one that fluctuates according to the negotiated power, process and relationships [47]. The progress needs to stay visible, offering pragmatic, actionable lessons can support this process and dialogue [45]. In order to move forward, we need to look further into participatory action research methods [45, 46, 48].

An important element to consider is the added workload that an action research programme entails. Action research should not be considered an additional task; the structured analysis and search for innovative solutions are integral parts of a systems strengthening approach [34]. Nevertheless, performing action research has definite implications for the currently overextended health district teams, which are often engaged in daily patient care routines and are not equipped with sufficient means. Additionally, one must not forget the additional work one asks from community representatives and actors from civil society. Further reflections are needed on the limitations of voluntary work and on offering a potential package of benefits that might mitigate the time and effort spent.

### Limitations of the study

Given the short project implementation time, we carried out only one action research cycle. Therefore, our observations are somewhat preliminary. The short duration precluded the generation of potential bottom-up knowledge and its input into the policy-making process. Therefore, this study needs to be seen as an intermediary evaluation of an ongoing and continuous process.

Furthermore, it could be argued that only a limited number of interviews [12] were undertaken. Nevertheless, we achieved data saturation and we strengthened the analysis by triangulation and closely involving various actors. Through discussions and intermediate seminars on the project at ITM, critical reflections were shared.

We were aware that the position of the external researchers in the study might have influenced data collection and analyses. The inclusion of our partner institution, Enabel, the main implementer, may have influenced the analysis process and the outcome. Moreover, the selection of interviewees might have introduced a respondent bias. To reduce these potential biases, we adopted a constant reflection attitude through systematic, rigorous documentation of the data collection and analytical processes as well as regular discussions with the members of the AI-PASS programme team and experts at ITM and the partner institutions. We performed data triangulation and we combined various data sources to mitigate these limitations.

## Conclusion

With this study, we showed that the action research approach can contribute to strengthening health systems. Understanding how and why services perform (or do not perform) well and considering the complexity of the system as a whole is critical to providing guidance for strengthening the functionality of those services in the long run. Certain dynamics are generated with this inclusive, participatory approach, with a specific focus on front-line workers. In this study, we found that creating a learning organisation, through intensive capacity-building and empowerment could enhance dialogue and ownership at the local level when certain conditions are met. Increased efficiency and responsiveness of the policy formulation and implementation process at higher level seems challenging. Strong linear, hierarchical structures, current work ethics and broader sociocultural aspects were found to be constraints. Conditions of utmost importance were sufficient time to permit actors to understand and engage in change, the identification and involvement of key actors, maintenance of a constant dialogue, and a pragmatic, flexible approach.

This study is part of an ongoing evaluation. Through documentation and reflections, the action research approach will be further analysed and adapted continuously over the coming years.

Although we presented a number of challenges, we also pointed to the potential of creating similar enabling environments. Further research is needed on the positive impact and possible constraints of the action research approach for guiding health systems management and policy. Therefore, this study advocates setting up reflective and participatory collaborations that encompass the double aim of initiating social change and gaining new knowledge.

## Supplementary information

**Additional file 1.** Interview guide.

**Additional file 2.** Main results of the local health system analysis in the two districts.

**Additional file 3.** Examples of Theories of Change developed in the two districts.

## Data Availability

The datasets used and/or analysed during the current study are available from the corresponding author on reasonable request.

## Abbreviations

AI-PASS: Institutional Support for Health Sector Strengthening
e-HPSR: Embedded Health Policy Systems Research
ITM: Institute of Tropical Medicine
LHS: Local health system
