# Supplementary material for: Action research and health system strengthening: the case of the health sector support programme in Mauritania, West Africa
Source: Health Res Policy Syst. 2020 Feb 19;18:25. doi: 10.1186/s12961-020-0531-1 (PMC7031916; doi:10.1186/s12961-020-0531-1)
Supplement: Supplementary file 1 — Additional file 1. Interview guide. [file 12961_2020_531_MOESM1_ESM.docx]

**Additional file 1 : Interview guide**

This interview topic guide gives an overview of the main questions that will be asked in interviews. This guide can be used during individual interviews and/or during focus group discussions.

**Abbreviations:** NGO: non-governmental organization; LHS: Local health system; AR: action research; ToC: Theory of change; AI-PASS: Institutional support for health sector strengthening

| **Elements** | **Aims / Guiding questions** |
| --- | --- |
| **Introduction** | Provide an introduction to the interview and state the purpose of the study  Preserve confidentiality and anonymity within possible limits  Seek permission to perform the interview (i.e., an informed consent form)  Seek permission to record the interview and/or take notes |
| **General section** | Confirm the background of the individual   - Public sector / private sector / NGO / Civil Society / Community - Central, regional, or local level - Degree or position (medical doctor, nurse, midwife, technical agent, auxiliary nurse, support personnel, other) |
| **Main questions** |  |
| LHS Analysis - content | Q: How would you describe the type and level of your participation during the LHS analysis process?   - Performer of the analysis: Yes/No - Member of the research team: Yes/No - Member of the AR Steering Committee: Yes/No - Participated in the analysis process through a workshop, interview, focus group discussion, or visit: Yes/No - Participated in the LHS and/or ToC workshop in October 2018: Yes/No   Q: How did you appreciate the use of the LHS model? In your opinion, were all the dimensions of the LHS model adequately clarified? *(Please list any elements that remain unclear)*  Q: In your opinion, what are the particular features of such an analysis? *(Prompt: What are the differences compared to a vertical project, a non-linear approach, or a participatory, cyclical, integrated system, etc.)*  Q: In your opinion, were the main challenges and weaknesses described during the analysis?  Q: Are there elements missing in the LHS analysis? If so, what are they?  Q: Are there elements that seem to be less (or not) useful in the LHS analysis? If so, what are they? |
| Process of analysis and workshops – the participatory approach | Q: How did you appreciate the program’s approach? *(Prompt: The purpose of the analysis and the program is to use a participatory approach by interacting with all stakeholders, including local actors and community members, etc.)*  Q: In your opinion, were the principal actors involved? If not, which actors did not participate sufficiently ?  Q: In your opinion, what are the strengths and weaknesses of the program’s approach? |
| Process of change | Q: What are, in your opinion, the most difficult aspects of creating change?  Q: What would you recommend? What kind of strategies would you propose or envision to stimulate change? |
| LHS analysis – future applications | Q: Do you think this methodology would be applicable in other contexts in Mauritania?  Q: What would you recommend? |
| AI-PASS program– double anchorage | Q: How did you appreciate the approach of the AI-PASS program? *(Prompt: the combination of central and operational support)*  Q: What are or could be possible threats or risks to the program? What elements should be taken into account?  Q: What are your recommendations for the future course of the project? |
| **End** | Thank the person for his/her participation  Confirm the preservation of confidentiality |
