# Supplementary material for: Action research and health system strengthening: the case of the health sector support programme in Mauritania, West Africa
Source: Health Res Policy Syst. 2020 Feb 19;18:25. doi: 10.1186/s12961-020-0531-1 (PMC7031916; doi:10.1186/s12961-020-0531-1)
Supplement: Supplementary file 2 — Additional file 2. Main results of the local health system analysis in the two districts. [file 12961_2020_531_MOESM2_ESM.docx]

**Additional file 2: Main results of the local health system analysis in the two districts**

***Dar Naim***

The district of Dar Naim is located in the Northern region of Nouakchott, the capital of Mauritania. It is a mixed urban and peri-urban area, which extends into precarious quarters built on sand dunes. The population grew from 61,089 to 173,663 inhabitants in 15 years (1,2). Overall, the community was poor: 20% of the inhabitants lived below the poverty level (3). Sources of income included small businesses in the formal and informal sectors (4). The population was a diverse community that comprised various tribes (Moors, Fula (Peul), Soninke, Wolof).

The health system was mixed and dynamic: two structures were run by the public sector, one structure was run by the local government; and others were funded and managed by private not-for-profit (N=8) and for-profit agencies, including (clinics (N=10), pharmacies (N=40) and traditional practitioners (N=4)), with little or no coordination. The health district was run by one medical officer with little means and no supporting district team. The plethora of human resources was unevenly distributed, and they lacked coaching and career planning. Analysis of the offer in healthcare showed a mismatch between the expressed needs and the supply of care. Quality was limited due to lack of resources in terms of drugs, equipment and logistical means on one hand, and lack of patient centered care and discontinuity on the other hand. Pharmacy management was inadequate as little training nor tools were made available. Analysis of financial management was challenging, due to lack of existing tools, but moreover due to lack of transparency, that additionally increased the level of dissatisfaction among the health workers.

Community participation was generally weak, especially due to the limited representation of communities in the dysfunctional management committee of the cost recovery system. Additionally, no platform for discussion or dialogue between health care services and the population existed.

On the other hand, the existence of some projects, run by local civil society organizations and sometimes supported by international actors (Memisa, Institute of Tropical Medicine), were observed with interest and showed potential for further development. A voluntary sickness fund and an equity fund, that strived for social protection, were functioning, but experienced some difficulties to survive.

In general, national policies didn’t guide the health district sufficiently towards a better quality of care, as little was adapted to local needs. The analysis showed that a new model of care that could be adapted to the urban context was of dire importance.

**Figure 1: Map of the main actors in Dar Naim**


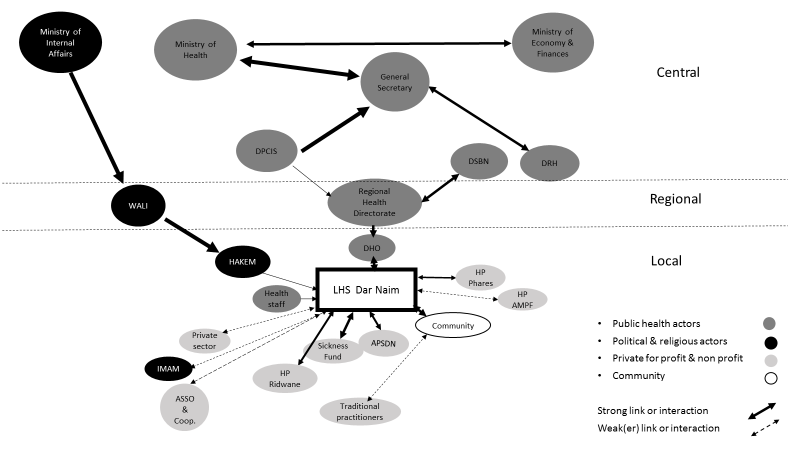


**Abbreviations:** LHS: Local Health System; DPCIS: Directorate of Planning of Cooperation and Health Information; DSBN: Directorate of Basic Healthcare and Nutrition; DRH: Directorate of Human Resources; DHO: District Health Officer; HP: Health Post; ASSO & Coop: Associations & cooperatives; APSDN: Association pour la Promotion de la Santé de Dar Naim; AMPF: Association Mauritanienne pour la Promotion de la Famille.

***Bababé***

The district of Bababé is a rural area, located in the South-western part of Mauritania, along the border of the Senegal river. The population was estimated to be 37,494 (2). The overall poverty rate was high, i.e. 43.3% (versus 31% at national level) were living below the poverty level (3). Sources of income included agriculture, cultivation, and small businesses. The literacy rate was low, particularly among women (0.56%). The population comprised mostly Fula and Moor tribes.

The district of Bababé was confronted with a huge problem, in terms of health care coverage, which greatly impacted the accessibility to health-care structures. Utilization rates of the health structures fluctuated between 0.2 and 0.7 new consultations/inhabitant/year; antenatal care coverage was below 50%, except in a few structures. The system included 13 public structures in total, with a few private clinics, and a high, but unknown number of traditional practitioners. The organization of care in and between the first and second levels of care faced substantial weaknesses. Weak complementarity and a poor referral system hampered access.

The lack of human resources and the inadequate technical platform for healthcare delivery led to a failure to meet the standards of quality of care. General fatigue and a low level of motivation was observed among most healthcare workers. Little attention was given for patient centered care. The healthcare package didn’t meet the standards defined at national level, in most of the structures.

The absence of a functional health district team led to the lack of adequate planning, activity coordination, and staff coaching. Most of the health district team members had never visited the health posts. Technical supervision was possible due to the support of a local NGO. The quality of the health information system seemed inadequate, not allowing an adequate analysis and limiting the definition and / or adaptation of strategies.

As in the other district, pharmacy management was weak and managed by a nurse assistant. Several misconducts were reported. Financial management was again not easy to assess due to lack of transparency and tools.

Apart from a few exceptions in some communities, the interface between the community and professional health actors was quite poor. Local initiatives were based on individual and/or community needs and often financed by foreign diaspora (as for example the construction of or donation of equipment for a health post), but were never guided by a dialogue with the health district manager or team.

**Figure 2: Map of the main actors in Bababé**


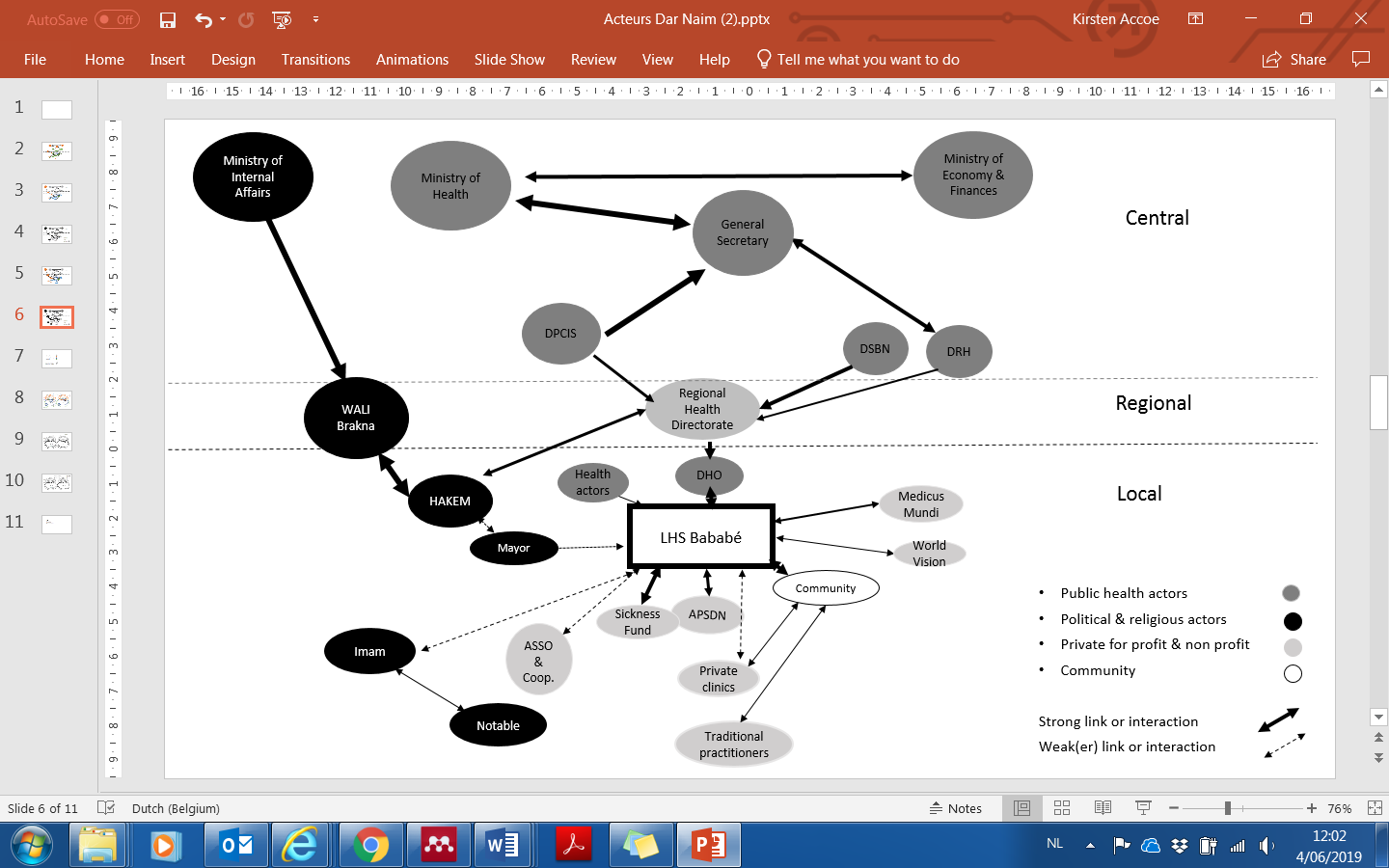


**Abbreviations:** LHS: Local Health System; DPCIS: Directorate of Planning of Cooperation and Health Information; DSBN: Directorate of Basic Healthcare and Nutrition; DRH: Directorate of Human Resources; DHO: District Health Officer; ASSO & Coop: Associations & cooperatives; APSDN: Association pour la Promotion de la Santé de Dar Naim.

As shown in Figure 1 and

Figure **2**, both districts had a parallel governance system. In principle, the central and regional directors of the Ministry of Health regulated and followed up all health activities. In practice, political actors (the Ministry of Internal Affairs, the Wali (governor), and the Hakim (prefect)) played prominent roles, especially at rural level. This was exemplified by the resource allocation, where the political influence dominated. Additionally, our analysis showed that socio-cultural factors played important roles in the interactions between the various actors.

***References***

1. Office National de la Statistique, Bureau Central du Recensement. Récensement Général de la Population et de l’Habitat. Répartition spatial de la population. Nouakchott; 2013. p. 28.

2. Ministère de la Santé République Islamique de Mauritanie, Direction de la Programmation de la Coopération et de l’Information Sanitaire. Intervention targets and demographic data 2017-2020. Nouakchott; 2017.

3. Office National de la Statistique. Enquête Permanente sur les Conditions de Vie des ménages. Nouakchott; 2014.

4. Office National de la Statistique, Bureau Central du Recensement. Monographie regionale de la wilaya de Nouakchott. Nouakchott; 2013. p. 1–60.
