# Supplementary material for: Action research and health system strengthening: the case of the health sector support programme in Mauritania, West Africa
Source: Health Res Policy Syst. 2020 Feb 19;18:25. doi: 10.1186/s12961-020-0531-1 (PMC7031916; doi:10.1186/s12961-020-0531-1)
Supplement: Supplementary file 3 — Additional file 3. Examples of Theories of Change developed in the two districts. [file 12961_2020_531_MOESM3_ESM.docx]

**Additional file 3: Examples of Theories of change of the two learning districts**

1. **Theory of change - Governance - Dar Naim**

**Desired situation:**

The Moughataa of Dar Naim has a functional district health team, composed of competent and motivated members, able to ensure an adequate coordination of the various stakeholders and benefiting from sufficient resources in order to strive for an adequate and autonomous management of the local health system.

**Theory of change - narrative:**

The change we wish to achieve, as described above, can be obtained through investment in four areas: the creation of a functional district health team, the development of an efficient, transparent and autonomous management system, the creation of an effective stakeholder coordination framework and the availability of resources. Each of these dimensions are depicted within a non-linear pathway wherein multiple interactions exist.

In order to create a functional district health team, it is necessary to define the roles, motivate the members and reinforce their capacities respectively. This requires a certain level of advocacy at central level in order to develop a legal framework, and to ensure mentorship and efficient support. Capacity building activities should focus on managerial capacities, formative supervision, encouragement of dialogue and the setup of a functional monitoring system. Implementation conditions include motivation and stability of human resources, which are related to different factors: an adequate support of the regional directorate and other partners, transparency in management and availability of sufficient resources.

In order to develop an efficient, transparent and autonomous management system, it is not only necessary to assess skills and to train staff accordingly, but also to standardize management tools and promote the culture of vertical and horizontal accountability. In order to ensure adequate management, it is important to advocate at central level to recruit financial managers and develop tools and standard procedures. Defined conditions are among others the follow-up of procedures and measures in case of misconduct.

To ensure efficient coordination, it is noted that agreements must be drawn with the various partners and periodic meetings need to be organized. However, a certain level of shared power and trust are prerequisites.

The availability of adequate resources is correlated with the previous three dimensions: it requires a team that knows how to manage and plan activities, an efficient management system to monitor and a coordination framework in order to mobilize existing resources. While HR management is ensured at regional level, a certain level of decentralization is needed. In addition, the availability of adequate logistics and infrastructure with a corresponding technical platform are other factors contributing to the functionality of the health system.

**Figure 1: Theory of Change - Governance in Dar Naim (French)**


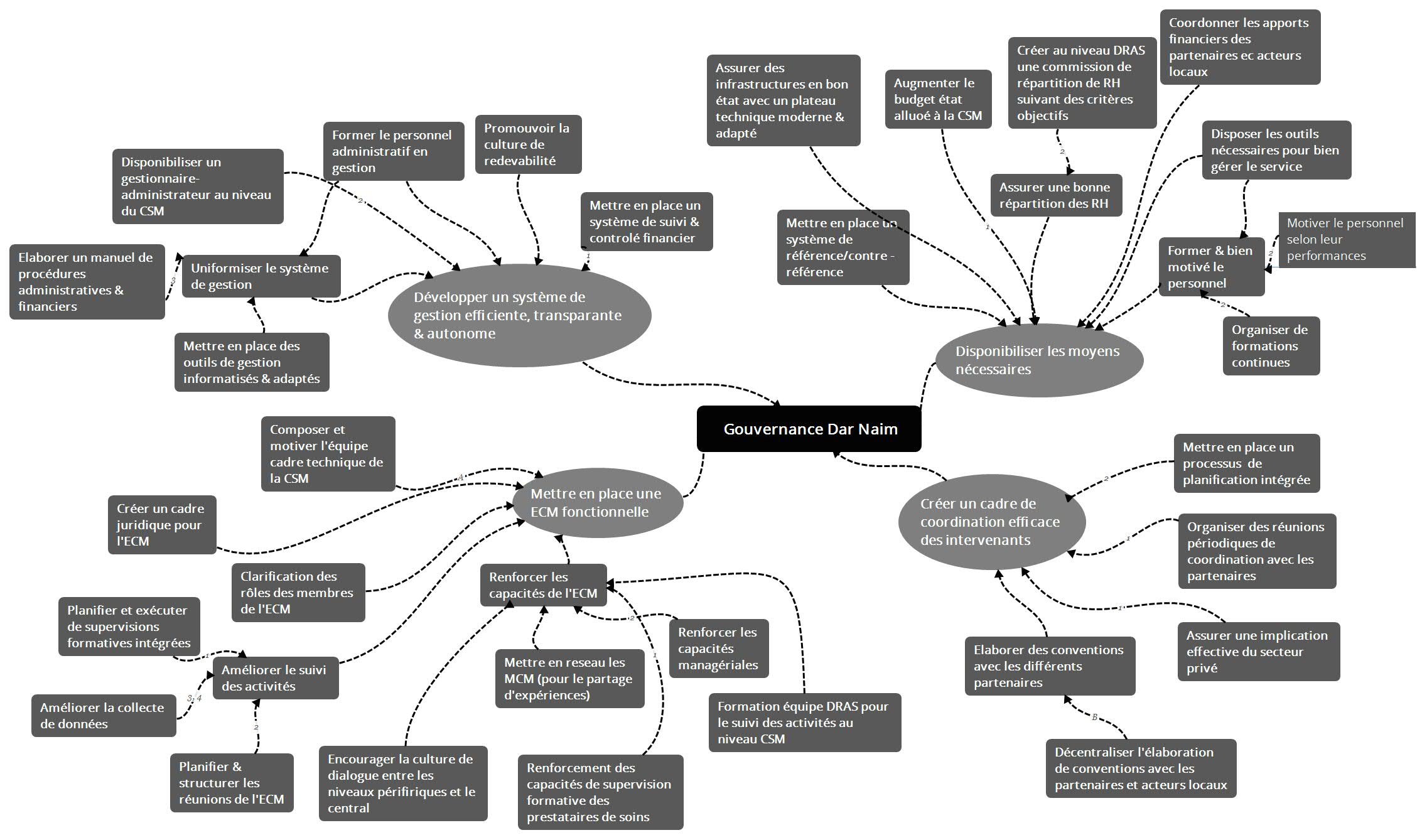


**Abbreviations :** CSM: Circonscription Sanitaire de la Moughataa ; ECM : Equipe cadre de la Moughataa ; MCM : Médecin Chef de la Moughataa

1. **Theory of change – Community Participation - Bababé**

**Desired situation:**

An active and inclusive community participation strategy supporting on:

- Functional community structures (health committees, basic health units, associations), adopting the principles of accountability and mutual valorization, serving as an interface between health facilities and the community; and
- A permanent dialogue between all local actors.

**Theory of change - narrative:**

The change we would like to obtain, as described above, can be achieved through investment in following main areas: the revitalization of community health strategies and health committees, the creation of a coordination platform and the promotion of a culture of dialogue, functioning as a red line throughout our strategy. To highlight: the community structures are tools to enhance community participation, but it is the act of listening, the communication skills and the values ​​of solidarity and equity that will contribute to the creation of a true partnership and will serve as a basis for the co-management of the local health system.

To create an improved dialogue, it is planned to promote the VCA approach (voice, citizen, action), to identify focal points to ensure a voice is given to the most vulnerable and to improve the dissemination of health information through, among others, local radio stations. This requires additionally for capacity-building of the district team in order to develop an open mind for dialogue and to create a climate of trust.

In order to create a coordination platform, it is recommended to invest in the strengthening and supervision of associations through support of partners, the launch of a website to improve national and international advocacy and the involvement of religious and local elected leaders.

The degree of functionality of health committees will depend on several elements, such as the redefinition of selection criteria allowing for contextualization and equity enhancement. The defined conditions include a fair selection process, a clear distribution of roles and responsibilities, and an adequate support from the district health team, the regional directorate and local leaders. Possible risks are lack of convergence of points of view and inter-community tensions. Ensuring that stakeholders are sufficiently informed, allowing for appropriate time and creating an atmosphere of dialogue and trust are strategies to be taken into account.

The revitalization of community health strategies includes three fundamentals: the functionality of basic health units and community health workers, the professionalization of the sickness fund and the inclusion of traditional healers. The availability of tools, a certain level of motivation and the development of training and supervision activities are pathways of change to consider in order to create functional basic health units. The stability of resources and the availability of quality drugs are other conditions to be taken into account. In relation to the sickness fund it is recommended to advocate for resources and capacity building to increase its capacity. The identification of a social worker in order to ensure the needs of the vulnerable population are respected, is a pathway to explore. Improved collaboration with traditional healers will require a mapping exercise in order to understand the context, but also in order to advocate for the valorization of the impact that this actor could have on patient-centered care. One risk is the lack of willingness of the Ministry of Health to integrate traditional medicine. Possible studies on traditional health practices may help.

**Figure 2: Theory of Change - Community Participation in Bababé (French)**


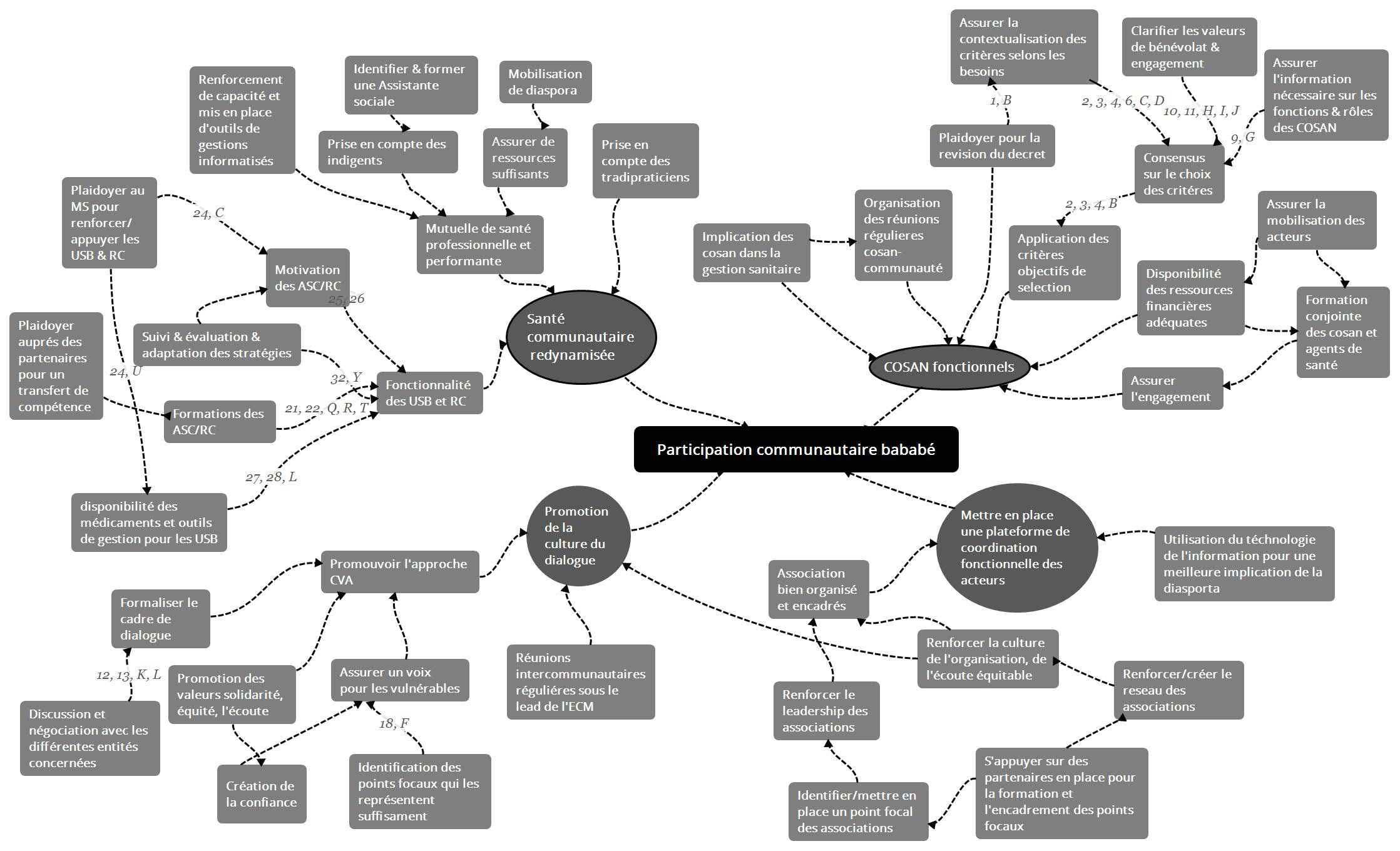


**Abbreviations :** MS: Ministère de la Santé; USB : Unité Santé de base ; RC : Relais communautaires ; CVA : Citizen – Voice- Action ; COSAN : Comité de Santé ; ECM : Equipe Cadre de la Moughataa
